# Two new species of *Edmockfordia* García Aldrete (Psocodea, ‘Psocoptera’, Epipsocidae), from Valle del Cauca, Colombia, and description of the female *E. chiquibulensis* García Aldrete

**DOI:** 10.3897/zookeys.503.9789

**Published:** 2015-05-11

**Authors:** Julián Alexander Mendivil Nieto, Ranulfo González Obando, Alfonso Neri García Aldrete

**Affiliations:** 1 Universidad del Valle, Departamento de Biología, Grupo de Investigaciones Entomológicas; 2 Universidad Nacional Autónoma de México, Departamento de Zoología, Instituto de Biología

**Keywords:** Neotropics, Belize, South America, Epipsocetae, taxonomy

## Abstract

Two new species of *Edmockfordia* García Aldrete, from Valle del Cauca, Colombia, and the female of *Edmockfordia chiquibulensis* García Aldrete, are described and illustrated. A key to the species of *Edmockfordia* is included; the genus was previously known only from Belize. The genus is re-diagnosed to include female characters. The distribution of the genus is considerably widened, from Belize to northeastern South America.

## Introduction

The genus *Edmockfordia* was described by García Aldrete (2009) on the basis of three male specimens collected with Malaise traps at the Chiquibul Forest Reserve, Belize; numerous additional specimens from the same locality have become available since, including females of *Edmockfordia chiquibulensis*. Two male specimens, representing each a different, undescribed species, were recently collected in Valle del Cauca, Colombia; the purpose of this work is to describe those two species and to re-diagnose the genus, to include female characters, besides, the distribution of the genus is extended from Belize to northern South America.

## Material and methods

The Colombian specimens available for study were collected with led light traps, at the Pericos Natural Reserve and El Danubio, in Buenaventura, Valle del Cauca, Colombia. The Belizean specimens were collected in flight interception traps, at the Chiquibul Forest Reserve, Cayo District, Belize. The specimens for microscopic examination were dissected in ethanol 80%, and their parts (head, right wings and legs, and genitals) were mounted on slides in Canada balsam, following the procedure in González et al. (2011). The whole specimens, before dissection, were placed in 80% ethanol and observed with a Nikon SMZ 645 microscope, for color description. Standard measurements were taken on the slides, utilizing a Nikon E200 microscope; the measurements are given in µm, and the abbreviations of parts measured are the following: FW, HW: lengths of right fore- and hind- wings, F, T, t1, t2: lengths of femur, tibia, and tarsomeres 1 and 2 of right hind leg, ctt1: number of ctenidobothria on t1 of right hind leg, f1…fn: lengths of flagellomeres 1...n of right antenna, IO, D, d: minimum distance between compound eyes, antero-posterior diameter and transverse diameter, respectively, of right compound eye, all in dorsal view of head, PO: d/D. The Colombian specimens are deposited in the Entomology Museum, Universidad del Valle (MUSENUV), Santiago de Cali, Colombia. The Belizean specimens are deposited in the Mexican National Insect Collection (CNIN), Instituto de Biología, UNAM, Mexico City.

## Taxonomy

### Edmockfordia calderonae sp. n.

Taxon classificationAnimaliaPsocodeaEpipsocidae

http://zoobank.org/0C1887DA-D8EA-4456-9A65-37F26DF77924

Figures 1–5

#### Type locality.

**COLOMBIA.** Valle del Cauca. Buenaventura. Vereda El Salto. Pericos Natural Reserve, 350 m., 3°56'N, 76°47'W.

#### Type material.

Holotype male, 9–11.VIII.2013. light trap, O. Saenz, N. Calderón. Deposited in the Entomology Museum, Universidad del Valle, Santiago de Cali, Colombia (MUSENUV slide No. 25775).

#### Etymology.

It is our pleasure to dedicate this species to Nadia Calderón, a graduate student at the Universidad del Valle, who, together with Oscar Saenz Manchola, collected the specimens of the two species of *Edmockfordia* here described.

#### Diagnosis.

Phallosome with three posterior projections, with posterior end broadly W shaped; external parameres short, little developed; aedeagal arch slightly projected posteriorly; epiproct broadly rounded posteriorly.

#### Color

(in 80% ethanol). Body mostly pale brown, with creamy areas, as indicated below, pronotum, propleura and metapleura brown, upper half of mesopleura brown, lower half creamy; meso- and metanotal lobes creamy, bordered with brown. Abdomen creamy, clunium brown. Head (Fig. 2), with a brown transverse band between compound eyes enclosing the ocellar triangle; vertex creamy; genae brown; ocelli hyaline, with dark brown centripetal crescents; postclypeus brown, anteclypeus centrally brown, sides creamy; labrum pale brown anteriorly, fading towards the posterior margin. Antennae with scape and pedicel brown, flagella pale brown. Maxillary palpomeres 1–4 brown. Legs: front coxa, and trochanters of all legs creamy; coxae of mid and hind leg with a dark brown spot on outer border; femora mostly creamy, with a brown spot on proximal and distal ends; tibiae and tarsi pale brown. Forewings hyaline, pterostigma with a brown spot distally, and a brown band proximally; veins brown, each with a brown spot distally, at wing margin; a brown spot at nodulus. Hindwings hyaline, veins pale brown, with brown spots distally, at wing margin. Epiproct and paraprocts creamy. Hypandrium creamy, with postero-lateral corners brown.

**Figures 1–5.**
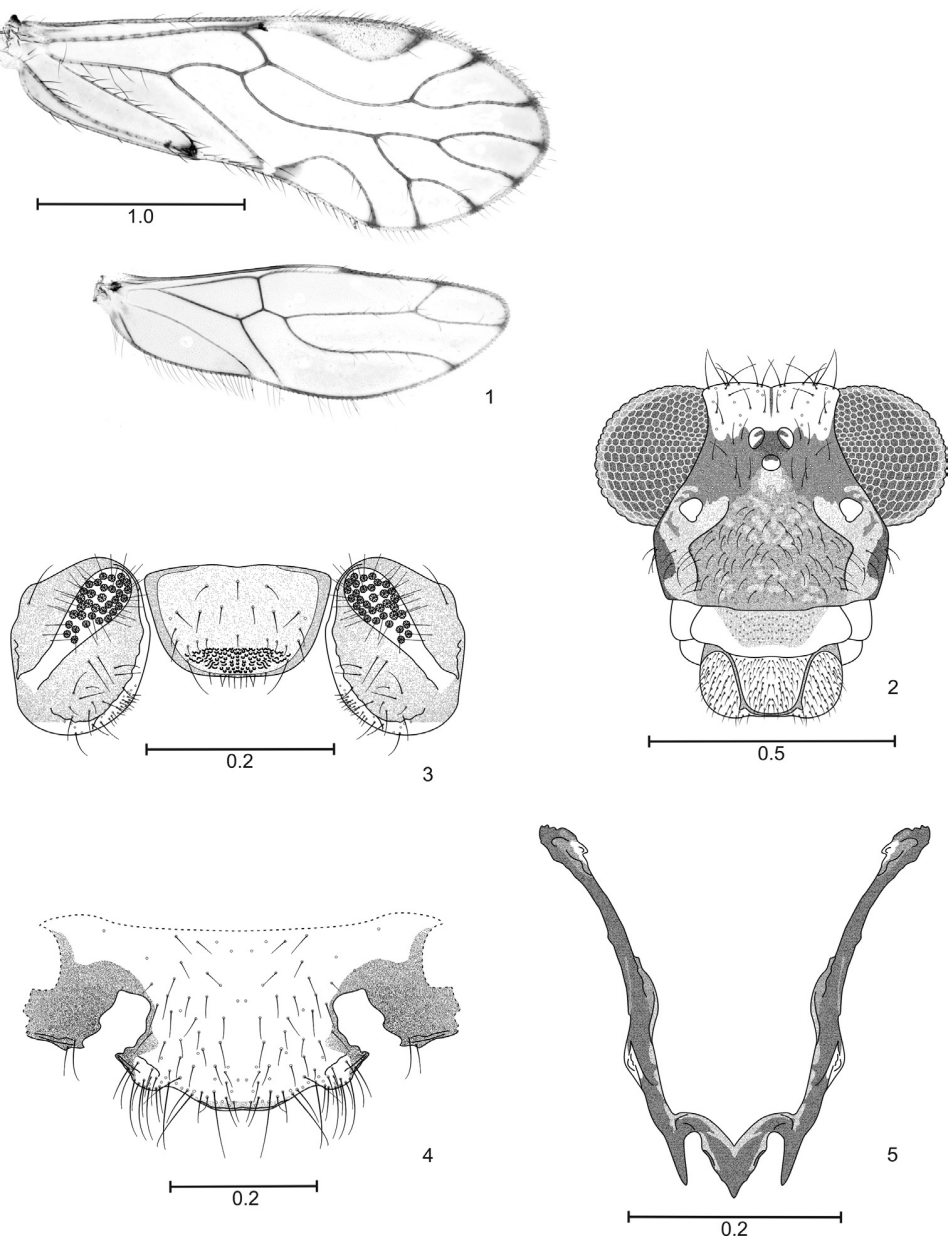
*Edmockfordia calderonae* sp. n. Male. **1** Forewing and hindwing **2** Front view of head **3** Epiproct and paraprocts **4** Hypandrium **5** Phallosome. Scales in mm.

#### Morphology.

As in diagnosis, plus the following: outer cusp of lacinial tip broad, with eight denticles; compound eyes with interommatidial setae, mostly dorsally. Forewings (Fig. 1) symmetric, pterostigma elongate, rounded, widest in the middle; Rs with two branches, M dichotomously branched, resulting in four branches; areola postica low, apically rounded. Hindwings symmetric (Fig. 1), Rs of two branches, M simple. Coxae of hind leg without Pearman’s organ; trochanters of all legs dorsally with two long setae, tarsi of front legs without ctenidobothria, mid legs with 21 ctenidobothria on t1, t2 with two ctenidobothria. Hypandrium (Fig. 4): symmetric, setose, posterior border convex. Phallosome (Fig. 5): open anteriorly, side struts long, slender, sclerotized, external parameres short; aedeagal arch slightly projected posteriorly in the middle; endophallus membranous, without sclerites. Paraprocts (Fig. 3): elongate, ovoid, with short setae and a field of microsetae along inner margin, sensory fields with 22–23 trichobothria on basal rosettes. Epiproct (Fig. 3): trapeziform, broadly rounded posteriorly, setose, with a field of microsetae along posterior border; a field of papillae mesally, next to posterior border.

#### Measurements.

FW: 2675, HW: 2000, F: 625, T: 1125, t_1_: 500, t_2_: 120, ctt_1_: 30, f_1_: 550, f_2_: 440, IO: 260, D: 240, d: 300, IO/d: 0.86, PO: 1.25.

### Edmockfordia saenzi sp. n.

Taxon classificationAnimaliaPsocodeaEpipsocidae

http://zoobank.org/E827F942-11AA-4E20-93BB-F82C4B9DECB0

Figures 6–10

#### Type locality.

**COLOMBIA.** Valle del Cauca. Buenaventura. Vereda El Salto. Pericos Natural Reserve, 350 m., 3°56'N, 76°47'W.

#### Type material.

**Holotype** male, 28–29.III.2013. Light trap, O. Saenz, N. Calderón. Deposited in the Entomology Museum, Universidad del Valle, Santiago de Cali, Colombia (MUSENUV, slide code No. 25774).

#### Paratypes.

2 male, **COLOMBIA.** Valle del Cauca. Buenaventura. Vereda El Danubio, 340 m., 3°36'58.8"N, 76°53'59.5"W. 28–29.VIII.2014. Light trap. R. González, O. Saenz, N. Calderón. Paratypes deposited in the Entomology Museum, Universidad del Valle, Santiago de Cali, Colombia (MUSENUV, slide code No. 26135–26136).

#### Etymology.

We take pleasure to dedicate this species to Oscar Saenz Manchola, one of its collectors, a graduate student at the Universidad del Valle, Santiago de Cali, Colombia.

#### Diagnosis.

Differing from *Edmockfordia calderonae* and from *Edmockfordia chiquibulensis* in having the phallosome aedeagal arch apically rhomboid, in having the external parameres extremely long, falcate, reaching the level of the aedeagal apex, and in having the postero-lateral corners of the epiproct rounded, protuberant.

#### Color

(in 80% ethanol). Body mostly brown, with creamy areas, as indicated below, pronotum and propleura brown; meso- and metanotal lobes creamy, bordered with brown, upper halves of meso- and metapleura brown, lower halves creamy. Abdomen creamy. Head (Fig. 7), with a broad, transverse, dark brown band between compound eyes, enclosing the ocellar group; vertex creamy; genae proximally creamy, distally brown; ocelli hyaline, with ochre centripetal crescents; postclypeus brown, anteclypeus brown in the center, creamy on the sides; labrum creamy. Antennae with scape and pedicel brown, flagella pale brown. Maxillary palpomeres 1–4 brown. Legs: coxae and trochanters creamy; femora mostly creamy, with a brown spot on proximal and distal ends; tibiae and tarsi pale brown. Forewings hyaline, pterostigma with a brown spot at the apex, and a brown band proximally, veins pale brown, each with a brown spot distally, at wing margin, a brown spot at confluence of Cu_2_ and A. Hindwings hyaline, veins pale brown, each with a brown spot distally, at wing margin. Clunium brown, epiproct pale brown, with sides dark brown; paraprocts pale brown, hypandrium almost unpigmented.

**Figures 6–10.**
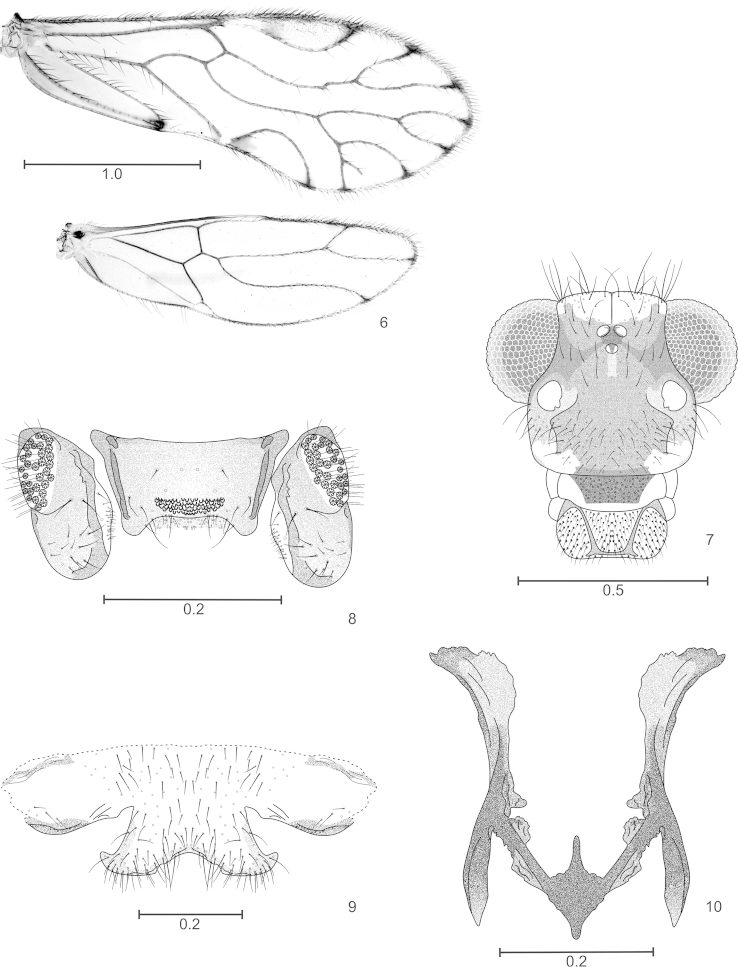
*Edmockfordia saenzi* sp. n. Male. **6** Forewing and hindwing **7** Front view of head **8** Epiproct and paraprocts **9** Hypandrium **10** Phallosome. Scales in mm.

#### Morphology.

As in diagnosis, plus the following: outer cusp of lacinial tip broad, with seven denticles; compound eyes with inter-ommatidial setae, mostly dorsally. Forewings (Fig. 6) asymmetric, pterostigma elongate, rounded, widest in the middle; Rs two branched; M dichotomously forked, resulting in four branches, right wing with M_4_ incompletely forked; areola postica low, apically rounded; left wing with open areola postica, vein Cu1A arising from M. Hindwings (Fig. 6) symmetric: Rs of two branches, M simple. Coxae of hind leg without Pearman organ, trochanters of all legs dorsally with two long setae; tarsi of front legs without ctenidobothria, t1 of mid legs with 11 ctenidobothria, t2 without ctenidobothria. Hypandrium (Fig. 9), symmetric, setose, with posterior border obtusely concave. Phallosome (Fig. 10): open anteriorly, V-shaped, side struts broad, dilated proximally and curved outwards. Paraprocts (Fig. 8): elongate, ovoid, with short setae, and a field of microsetae along the inner border, sensory fields with 30–31 trichobothria on basal rosettes. Epiproct (Fig. 8): broad, trapeziform, with field of short setae and a field of microsetae along posterior border; a field of papillae mesally, next to posterior border.

#### Remarks.

**Figure 11.**
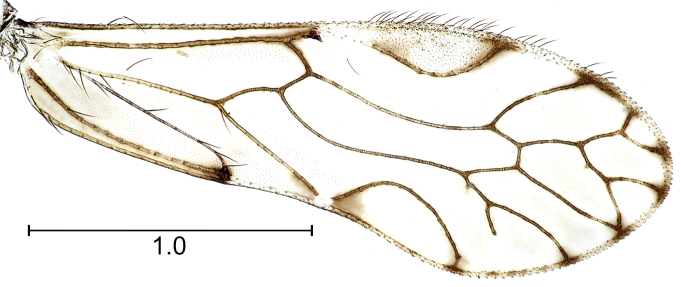
Anomalous forewing venation in *Edmockfordia saenzi* sp. n. Scale in mm.

Of the two paratypes for this species, one of them presents a forewing vein pattern with a four branched M, as described for the genus, however, a second specimen present the distal third of the forewing reticulated (Fig. 11) (e. g. R4+5 and M1 connected by a cross vein, R4+5 distally forked, a vein arising from the middle of R4+5 forked at wing margin, with the second branch fused at wing margin with M1, forming a closed cell, M1 absent, and M3 branched, with the branch near the areola postica incomplete.

#### Measurements.

FW: 2700, HW: 2025, F: 675, T: 1150, t_1_: 500, t_2_: 140, ctt_1_: 26, f_1_: 540, f_2_: 470, IO: 280, D: 180, d: 280, IO/d: 1, PO: 1.5.

### Edmockfordia chiquibulensis

Taxon classificationAnimaliaPsocodeaEpipsocidae

García Aldrete, 2009

Figures 12–16

#### Color

(19 years, three months in 80% ethanol). Essentially as in the male (see García Aldrete 2009). Scape and pedicel brown, flagella pale brown. Legs pale brown.

#### Morphology.

**Figures 12–16.**
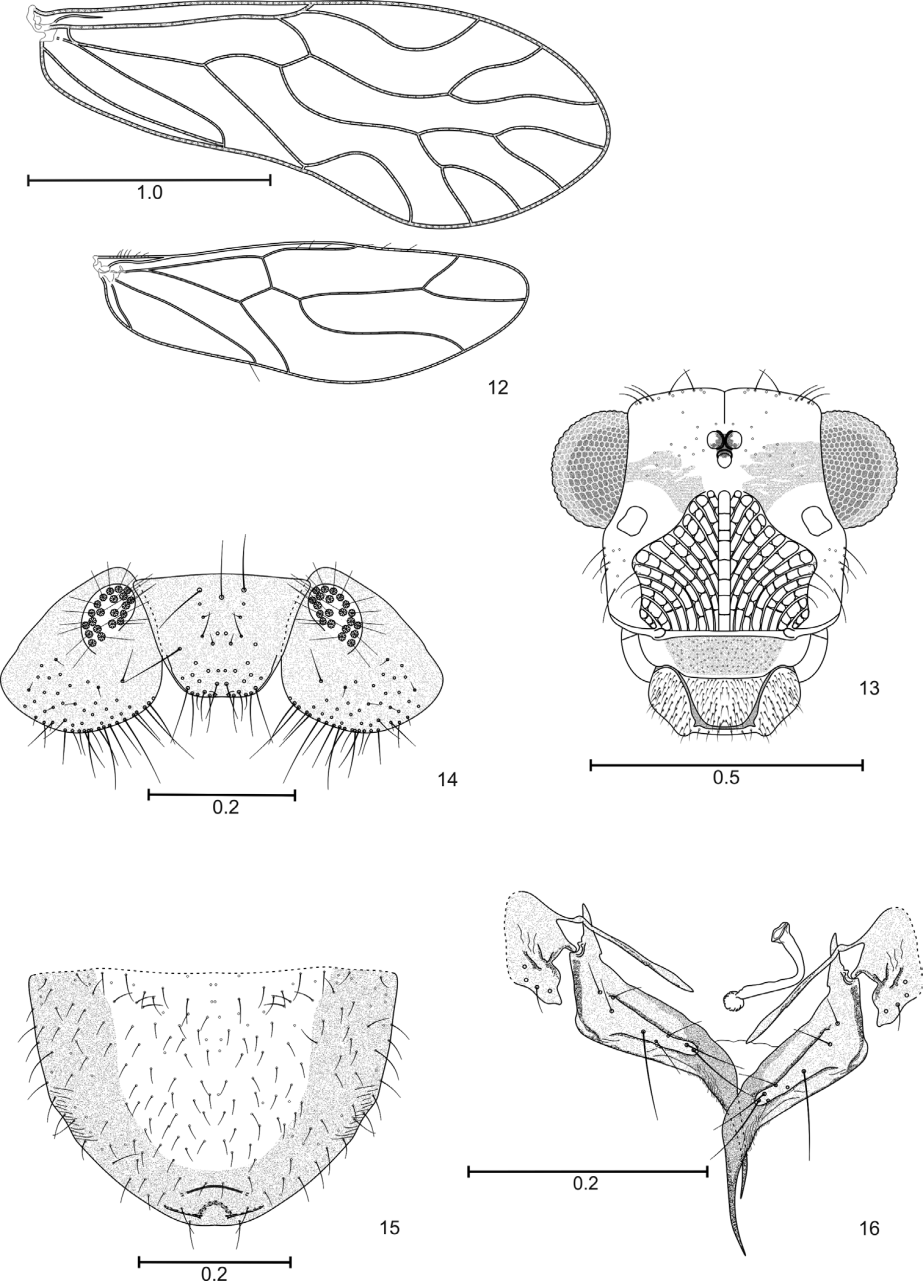
*Edmockfordia chiquibulensis* Female. **12** Forewing and hindwing **13** Front view of head **14** Epiproct and paraprocts **15** Subgenital plate **16** Gonapophyses and IX sternum. Scales in mm.

Wings (Fig. 12), distal labral sensilla and pretarsal claws as in the male (see García Aldrete 2009). Outer cusp of lacinial tip with seven denticles. Labral sclerites joined distally by a sclerotized band (Fig. 13). Subgenital plate (Fig. 15) broad, with field of short setae; pigmented area deeply concave anteriorly; posterior border rounded, a strongly sclerotized, concave band next apex, and a transverse crease anterior to it. Gonapophyses (Fig. 16) complete, v1 slender, about half the length of v2+3, joined to clunium by a membranous band. v2+3 wide based proximally, with a short, pointed “heel”; v3 a well defined, long slender lobe on side of v2, bearing eight setae; distal process almost straight, acuminate, with a field of microsetae proximally. Paraprocts (Fig. 14) broadly semicircular, with setae as illustrated, and a field of microsetae next distal border. Sensory fields elliptic, with 21 trichobothria on basal rosettes. Epiproct (Fig. 14) trapeziform, with field of setae on distal half, and three mesal macrosetae next anterior border.

#### Measurements.

FW: 2434, HW: 1784, F: 555, T: 998, t1: 449, t2: 113, ctt1: 27, f1: 608, f2: 493, IO: 343, D: 227, d: 159, Mx4: 184, IO/d: 2.15, PO: 0.46.

#### Specimens studied.

Belize. Cayo District. Chiquibul Forest Reserve. New María, 24.III.1995, 640 m. Malaise trap, paratype female. 17–20.I.1995, flight interception trap, female paratype. San Pastor, 560–580 m. 23–26.III.1995, paratype female. All specimens collected by T. King & A. Howe.

### A new diagnosis of *Edmockfordia* García Aldrete

Belonging in the Epipsocidae. Five distal labral sensilla, one central placoid, flanked at a distance by a pair trichoid-placoid. Without row of cuticular cones on setal bases of fore- femora. Forewings Rs 2 branched, M dichotomously branched, resulting in 4 M veins, Hindwings Rs 2 branched, M unbranched. Phallosome open anteriorly, broadly V-shaped, with side struts stout, proximally curved outwards; external parameres well developed. Aedeagal arch projected posteriorly, or rhomboid in the middle. Endophallus membranous, without sclerites. Paraprocts with a sclerotized marginal band, next inner border. Epiproct trapeziform, with a field of papillae mesally, next posterior border. Female subgenital plate with a concave sclerotized band next apex. Gonapophyses complete, v1 short, slender, v2+3 with a proximal heel.

### Key to the species of *Edmockfordia*

**Table:** 

1	Phallosome with one posterior projection, external parameres long, well developed (Fig. 10)	**2**
–	Phallosome with three posterior projections, external parameres short, little developed (Fig. 5)	***Edmockfordia calderonae* sp. n.**
2	Posterior projection of phallosome triangular, external parameres not reaching the posterior level of the aedeagal arch	***Edmockfordia chiquibulensis* García Aldrete**
–	Posterior projection of phallosome rhomboid, external parameres long, reaching the posterior level of the aedeagal arch (Fig. 10)	***Edmockfordia saenzi* sp. n.**

## Discussion

The species here dealt with extend the distribution of *Edmockfordia* from the Chiquibul Forest Reserve, in Belize, to Valle del Cauca, Colombia, all across Central America to northern South America. The two new species confirm the diagnosis of the genus: vein M in forewing dichotomously branched, side struts of phallosome stout, curved anteriorly, external parameres conspicuous, aedeagal arch projected posteriorly, paraprocts with a sclerotized band along inner border, and epiproct trapeziform, bearing a field of papillae mesally, next to the posterior border. The three species known in the genus differ in genitalic details, as indicated in the key above.

## Supplementary Material

XML Treatment for Edmockfordia calderonae

XML Treatment for Edmockfordia saenzi

XML Treatment for Edmockfordia chiquibulensis
